# Supplementary material for: Total Flavonoids Extracts of Apocynum L. from the Ili River Valley Region at Different Harvesting Periods and Bioactivity Analysis
Source: Molecules. 2022 Oct 28;27(21):7343. doi: 10.3390/molecules27217343 (PMC9655940; doi:10.3390/molecules27217343)
Supplement: Supplementary file 1 [file molecules-27-07343-s001.zip › Supplementary Figure S1.pdf]

Supplementary Figure

A

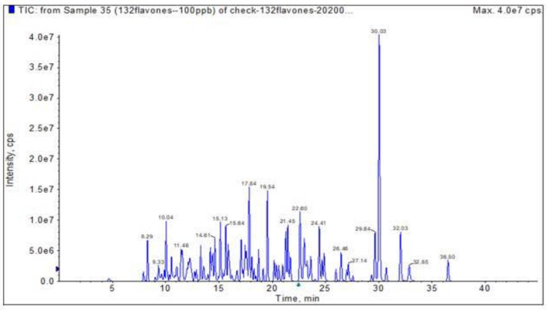

B

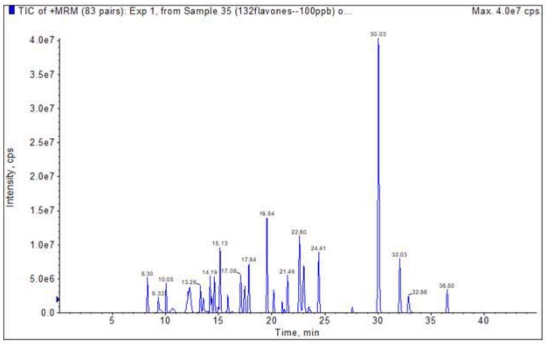

C

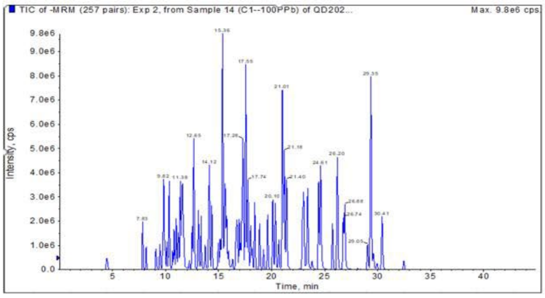

D

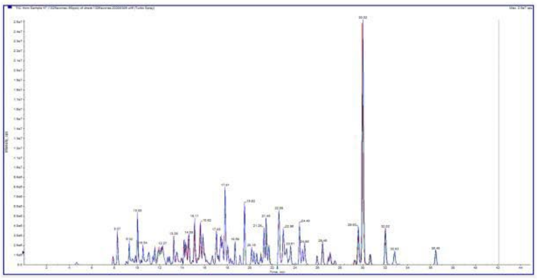

E

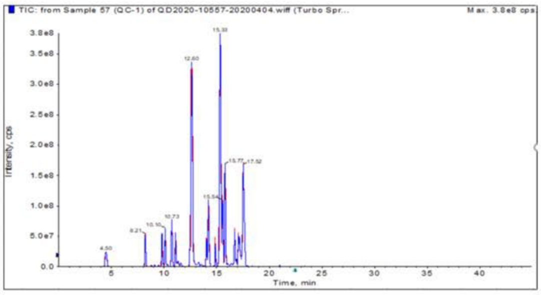

Figure S1. Total ions current plot for standards (**A**), TIC plot for standards in positive ionization mode (**B**), TIC plot for standards extraction in negative ionization mode (**C**), TIC overlay plot of standards (**D**), TIC overlay plot of quality control samples (**E**).
